# Dealing With the Pandemic of COVID-19 in Portugal: On the Important Role of Positivity, Experiential Avoidance, and Coping Strategies

**DOI:** 10.3389/fpsyg.2021.647984

**Published:** 2021-06-24

**Authors:** Maria José Ferreira, Rui Sofia, David F. Carreno, Nikolett Eisenbeck, Inês Jongenelen, José Fernando A. Cruz

**Affiliations:** ^1^HEI-Lab: Digital Human-Environment Interaction Lab, Faculty of Psychology, Education and Sports, Lusófona University, Porto, Portugal; ^2^Center of Research, Education, Innovation and Intervention in Sport, Faculty of Sport, University of Porto, Porto, Portugal; ^3^School of Sports and Leisure, Polytechnic Institute of Viana do Castelo, Viana do Castelo, Portugal; ^4^Department of Psychology, University of Almería, Almería, Spain; ^5^Department of Personality, Evaluation and Psychological Treatment, University of Seville, Seville, Spain; ^6^School of Psychology and Life Sciences, Lusófona University, Lisbon, Portugal; ^7^CiPsi – Psychology Research Center, University of Minho, Braga, Portugal

**Keywords:** existential positive psychology, acceptance, experiential avoidance, positivity, well-being, psychological distress, coping, COVID-19 pandemic

## Abstract

The global COVID-19 pandemic crisis has caused an unprecedented impact on most areas of people’s lives. Thus, framed within the scope of Existential Positive Psychology (PP2.0), this study aimed at assessing the psychological distress of adults living in Portugal during the first national lockdown, how they are coping with stress, as well to contribute to a deeper understanding about the role that positivity, experiential avoidance, and coping strategies have in psychological distress and well-being. For this purpose, 586 Portuguese adults (73% females) ranging between 18 and 78 years old (*M* = 38.96, *SD* = 12.20) completed an online survey during the initial phase of the pandemic crisis in Portugal. Findings suggest that experiential avoidance was the strongest predictor of a negative response (depression, anxiety, stress, loneliness, and negative emotions), whereas positivity was a better predictor of psychological well-being and lower levels of depression. Additionally, self-blame, behavioral disengagement, and emotional venting were strong risk factors for psychological distress, whereas positive reframing, planning, and acceptance were associated with more positive outcomes. These findings highlight the critical role of experiential avoidance on individuals’ psychological distress and the essential contribution of positive life orientation in promoting flourishing. By offering a better understanding of the complex navigation through the dialectics between positive and negative life features, this study provides important and useful cues for psychological interventions directed at promoting a more positive and adaptive human functioning even through such potential adverse and painful life events.

## Introduction

The pandemic crisis of COVID-19 is currently the more threatening situation most individuals have ever experienced. This crisis has caused an unprecedented impact on most areas of people’s lives, along with the uncertainness regarding its duration and the ongoing personal and social adjustments to this new “way of life” (e.g., Liu et al., 2020; Rodríguez-Rey et al., 2020; Wang et al., 2020; Zacher and Rudolph, 2020). Therefore, individuals’ psychological functioning and the factors that increase psychological distress and promote well-being and adaption to the crises have been the main focus and priority of current research.

Over the last years, Positive Psychology has provided several essential contributions to studying the factors that promote flourishing. In contrast with the more traditional perspectives on mental health, which are almost exclusively focused on psychological problems and psychopathology, the positive psychology movement advocates a new research focus (Seligman and Csikszentmihalyi, 2000). Psychology should not restrict the study of individual problems and the solutions to such issues. Instead, it must identify and better understand the factors and conditions that promote a more positive human, social, and organizational development (Csikszentmihalyi, 2009; Csikszentmihalyi, 2011). This relatively new psychology field has already developed in continuous and overlapping waves of growing knowledge and complexity (Lomas et al., 2020). In the first instance, while attempting to “break” with the deficit-centered model, positive psychology essentially focused on the positive aspects of human functioning (first wave). More recently, a second wave moved positive psychology to a distinct period beyond simply compartmentalizing the positive and negative aspects of human experience, offering a more differentiated and integrated understanding of these aspects and the relationships between them. It assumes that individuals follow, along the way, various developmental trajectories, which are often non-linear. A set of favorable conditions may follow low or negative general functioning, and many unfavorable conditions may be followed by positive and adapted functioning (Nakamura, 2011). Existential Positive Psychology (also termed PP2.0) arises from the integration of the Existential Humanist Psychology with Positive Psychology, aiming to further understand how adverse circumstances and suffering can result in positive and adaptive functioning (Ivtzan et al., 2016; Lomas and Ivtzan, 2016; Wong, 2011, 2017). Therefore, this perspective intends to integrate the positive and negative features of human existence systematically. Undesirable, painful, and damaging events are an inevitable part of people’s lives. Indeed, they can often lead to personal growth and serve as promoters of meaning in life. There is considerable growing evidence suggesting that mental stability and well-being are more influenced by how individuals attribute meaning to their internal experiences than by their actual negative impact (e.g., how negative they are; Lyubomirsky, 2001). Under this perspective, Existential Positive Psychology calls for the adoption of a dialectical way of coping with life demands, that is, responding effectively to both negative and positive aspects of living (Wong, 2011, 2012; Wong et al., 2006).

The way a person relates to their negative emotions and thoughts has a significant impact on their well-being. A research area that has yielded extensive evidence in this direction is the psychological inflexibility literature. Psychological inflexibility refers to the rigidity in which people respond to life events. In their attempt to avoid painful internal experiences, they may reduce contact with the present moment. This avoidance is likely to be associated with acting less effectively in a situation and being overwhelmed by their uncontrollable and feared internal experiences. Psychological flexibility, in turn, is characterized by people’s ability to adjust their behavior to the situation, guided by their goals and values (Hayes et al., 2006). Acceptance and experiential avoidance are examples of psychological flexibility and inflexibility, respectively (Bond et al., 2011). Acceptance is not merely tolerance to face negative experiences and emotions; it is the active, non-judgmental embracing of the experience “here and now” (Hayes, 1994, 2004; Hayes et al., 2006). Moreover, it also involves exposure and an authentic experience of feelings, thoughts, and sensations. Conversely, when people cannot accept unpleasant experiences and emotions, change or control situational factors, they begin to avoid painful thoughts and feelings themselves (Hayes, 2004). These deliberate attempts to suppress thoughts and feelings can, paradoxically, increase their occurrence with a behavioral and psychological negative impact. This avoidance results in a series of deliberate efforts to control these feelings’ frequency, duration, and intensity, often leading to adverse outcomes already well documented in the literature (Hayes-Skelton and Eustis, 2020).

The central role that experiential avoidance plays in psychological health and well-being has been investigated in numerous studies (e.g., Gerhart et al., 2014; Spinhoven et al., 2014; Machell et al., 2015; Pierson et al., 2019). Previous research also found that experiential avoidance is related to depression (Moroz and Dunkley, 2019), anxiety symptoms (Zvolensky et al., 2016), and self-harm (Anderson and Crowther, 2012; Zvolensky et al., 2016; Brereton and McGlinchey, 2020). Higher experiential avoidance and avoidance coping seem to predict an increased lifetime frequency of self-harm (Nielsen et al., 2016). The influence of experiential avoidance in the psychological impact of the COVID-19 pandemic is also being explored. In a recent study with healthcare professionals, experiential avoidance was found to be a risk factor that exacerbates the adverse effects on psychological adjustment (Seçer et al., 2020). Results from additional research have consistently demonstrated that psychological inflexibility exacerbates the detrimental impacts of COVID-19 on mental health (Dawson and Golijani-Moghaddam, 2020; Kroska et al., 2020; Pakenham et al., 2020). Conversely, acceptance, or even embracing painful emotions and thoughts, and aligning actions to one’s core values have been associated with more adaptive outcomes (Lopez et al., 2020).

Beyond the importance of acceptance, adaptive coping with life demands requires positive orientations. Positive life orientation or positivity refers to the dispositional tendency to look at the current, past, and present life from a positive perspective and integrates the common features of self-esteem, life satisfaction, and optimism (Caprara et al., 2010; Alessandri et al., 2012a). In fact, since early studies, authors have identified a common latent factor in the three previously mentioned constructs, originally called “positive thinking” and, more recently, positive orientation or positivity (Caprara et al., 2010). Positive orientation emerges as a dispositional tendency that exerts a predominant influence on the way people look at life, fostering more “color” and understanding of its potentials more clearly. However, this life orientation does not necessarily imply that individuals protect themselves from the experience of negative emotions but rather tend to have a positive, realistic outlook on life experiences and attitudes toward the future. Positivity as a basic self−evaluative disposition seems to be a promising predictor of optimal functioning, as well as may act as a protective factor against mental illness (Alessandri et al., 2012b; Caprara et al., 2017, 2018). Current research has already established the critical role of psychological flexibility and positive life orientations, two dispositional characteristics, toward a better adjustment to life adversities (Trzebiński et al., 2020; Yıldırım and Güler, 2021).

Notwithstanding the great importance of these dispositional characteristics in successful adaptation, it is also essential to analyze the situational coping strategies that individuals use most frequently to deal with specific stressful situations. Analyzing the type of strategy and how they are associated with and contribute to better psychological and physical outcomes has been the focus of several studies (e.g., Babore et al., 2020; Dawson and Golijani-Moghaddam, 2020; Mariani et al., 2020; Rettie and Daniels, 2020; Jarego et al., 2021). Coping strategies refer to behavioral and cognitive efforts used by individuals in order to reduce the pressure of a stressful situation when its demands exceed personal resources (Lazarus and Folkman, 1984). Coping is seen as a dynamic system, depending on the interaction that triggers stress. Nevertheless, the coping literature is quite complex, and there is not enough consensus regarding the types of response and the consistency of these responses (Skinner et al., 2003). Lazarus and Folkman (1984) proposed the original coping model, which distinguished problem-focused coping strategies responses addressed to an external event and oriented to reduce or eliminate stress, and emotion-focused coping strategies, including responses adopted to control internal emotional reactions. When individuals feel that something constructive can be done, problem-focused coping usually prevails; on the contrary, when individuals feel that the stressor tends to remain, emotion-focused coping are more likely to be used. Additionally, avoidant strategies or disengagement coping is also another type of coping, which includes strategies used with the sole purpose of keeping undesirable emotions and thoughts out of conscious awareness (Billings and Moos, 1981). In the present pandemic crisis, avoidant coping responses, such as denial, behavioral disengagement, and self-distraction, partially mediated the relationship between intolerance of uncertainty and depression (Rettie and Daniels, 2020). Emotion-focused coping strategies seemed to increase anxious and depressive symptoms (Mariani et al., 2020), and social support and avoidance predicted distress levels in a sample of healthcare workers in Italy (Babore et al., 2020). In a recent study with Portuguese participants, coping strategies of positive reframing and humor predicted better mental health, whereas substance use predicted poorer mental health (Jarego et al., 2021). Despite the multiple studies that analyze the impact of using different coping strategies, each strategy’s degree of effectiveness and adaptation depends mainly on the context’s and individual’s characteristics. Therefore, it is not possible to categorize strategies as positive or negative, adaptive or maladaptive. It also depends on the perceived level of demand and the individual’s control of the situation and the stressor (Smith and Kirby, 2011). Usually, adaptive strategies would result in stress reduction or enhanced well-being and mental health outcomes. The widely varying results obtained in the latest studies conducted regarding the pandemic crisis and the dynamic nature of coping reinforce the need to further study how different coping strategies are associated with positive and negative outcomes.

Concerning individuals’ mental health status in the current pandemic crisis, recently published studies have reported a high prevalence of mental health problems in different populations throughout the coronavirus pandemic. Specifically, recent data concerning immediate psychological impact among Portuguese individuals suggest that participants reported higher levels of moderate to severe symptoms of depression, anxiety, and stress than previously (Passos et al., 2020; Paulino et al., 2020; Moreira et al., 2021). However, another study has observed normal mental health levels (Jarego et al., 2021) during the first lockdown. The authors discussed this result as possibly being influenced by the participants’ sociodemographic characteristics. However, these findings are cross-sectional, and thus it is not possible to establish a causal or direct effect of the pandemic. Some of these studies supported their conclusions by comparing the average pre-pandemic levels with the data collected during the first months of the pandemic. Despite the undeniable importance of such preliminary findings, longitudinal designs would provide a better understanding of the true impact of the pandemic on individuals’ mental health.

Moreover, a growing number of studies have generated some relatively consistent data regarding the differentiated impact of the pandemic according to some sociodemographic variables, such as gender and age. National and international studies in the early stages of the pandemic crisis indicated that the female gender is associated with a more significant psychological impact, reflecting higher levels of anxiety, stress, and depression (Paulino et al., 2020; Wanberg et al., 2020; Wang et al., 2020; Moreira et al., 2021), and PTSD (González-Sanguino et al., 2020). The COVID-19 pandemic and lockdown also seem particularly stressful for younger adults (Pieh et al., 2020). Older adults seemed to have a more optimistic outlook and better mental health during the pandemic’s initial stages (Bruine de Bruin, 2020) and better mental health outcomes (Smith et al., 2020). Conversely, income seems to have a distinct impact on different studies and countries. For example, among studies performed in the United Kingdom and United States, individuals at higher levels of income experienced a more significant decrease in life satisfaction (Wanberg et al., 2020) and reported a more detrimental impact on mental health (Daly et al., 2020). Conversely, in Spain, higher income was associated with a lower risk of depression (García-Álvarez et al., 2020), whereas in China, having a steady family income was a protective factor for college students against anxiety during the COVID-19 outbreak (Kontoangelos et al., 2020).

Regardless of the interest and importance of analyzing the psychological distress of the population affected by this pandemic crisis, it is also crucial to analyze some dispositional characteristics, such as experiential avoidance and positive life orientation, toward a more positive human functioning. The specific contribution of these variables in predicting both psychological distress and well-being in the context of the COVID-19 pandemic is still unknown. Besides, studies concerning the most effective coping strategies to deal with the pandemic are, as described previously, quite variable depending on the context and the group studied (e.g., Babore et al., 2020; Mariani et al., 2020; Rettie and Daniels, 2020; Jarego et al., 2021).

It seems, therefore, very useful to analyze the contribution of coping strategies, together with more dispositional measures, to different measures of psychological functioning such as psychological distress and well-being. The analysis of the relations between these variables, as well as understanding the role of psychological factors in buffering the effect of social context on psychological functioning should be a research priority to design effective longer-term strategic programs (Holmes et al., 2020).

In this sense, this study aims to:

(1) evaluate the levels of psychological distress of adults living in Portugal during the first national lockdown associated with the COVID-19 outbreak;
(2) analyze which coping strategies were more used among the participants of the current study to deal with de pandemic crisis, and finally;
(3) examine how positivity, experiential avoidance, and coping strategies are associated with well-being and psychological distress.

## Materials and Methods

### Participants

A preliminary analysis to calculate the minimum sample size recommended for detecting a significant effect in hierarchical multiple regression was performed using an online calculator. Thus, 20 predictors were considered for a medium effect size of 0.15 (Cohen’s *f*^2^), a power of 0.80 and an alpha level of 0.01 (Cohen, 1988; Soper, 2018). For this study, 194 would be the appropriate minimum of participants to allow the detection significant effects.

A total of 630 adults completed the online survey. Inclusion criteria were (a) being 18 years old or older; (b) living in Portugal at the time of the first national lockdown; (c) being able to read and understand Portuguese; and (d) after being informed of the objectives and conditions of the study, agreed to participate. Forty-four participants (6.98%) were excluded because they did not reside in Portugal at the moment of the study. Therefore, the final sample included 586 participants (72.9% female) ranging between 18 and 78 years old (*M* = 38.96, *SD* = 12.20). Full demographic characteristics are presented in Table 1. Most participants were married (40.6%), followed by single (25.3%), in a relationship (18.4%), in cohabitation (9.9%), divorced (5.6%), and widowed (0.2%). Additionally, participants’ level of education was as follows: bachelor or master’s degree (69.3%), high school (18.4%), Ph.D. (5.8%), and basic school – 9th grade (1.9%), among which 4.6% were still students. Regarding perceived social status, most considered themselves as medium (81.2%), whereas 8% perceived themselves as below medium and 10.8% above medium.

**TABLE 1:** Demographic characteristics of the participants.

Demographics characteristics	*n*	%
**Gender**		
Female	427	72.9
Male	159	27.1
**Marital status**		
Single	148	25.3
In a relationship	108	18.4
In cohabitation	58	9.9
Married	238	40.6
Widowed	1	0.2
Divorced	33	5.6
**Level of education**		
9th grade	11	1.9
High school	108	18.4
Bachelor or master’s degree	406	69.3
Ph.D	34	5.8
Sill a student	27	4.6
**Perceived social status**		
Below medium	47	8
Medium	476	81.2
Above medium	63	10.8

### Measures

#### Demographic Characteristics

Participants were asked to provide some demographic characteristics, including gender, age, education level, marital status, and perceived socioeconomic status.

#### Well-Being, Perceived Physical Health, Negative Emotion, and Loneliness

The PERMA – Profiler was used to assess these variables. This instrument was developed by Butler and Kern (2016) as a measure of general well-being, based on Seligman’s (2011) PERMA model of flourishing, which reflects a “dynamic optimal state of psychosocial functioning that arises from functioning well across multiple psychosocial domains” (Butler and Kern, 2016, p. 2). Thus, it includes the following subscales, reflecting the domains of the model: positive emotion (P; tendency to feel positive emotions), engagement (E; feeling interested and absorbed in an activity), relationships (R; expressing and receiving social support), meaning (M; feeling part of something greater than individual existence), and accomplishment (A; feelings of achievement and striving for goal attainment). The total score of these subscales constitutes a measure of overall well-being. Additionally, the PERMA also includes the subscales of negative emotion, perceived physical health, and loneliness. The total scale includes 23 items (e.g., *To what extent do you feel loved*?) rated on a 7-point scale from 0 to 6, instead of the original 11-point. The response scale was changed to be consistent with the rest of the questionnaires used in this study. A preliminary study on the psychometric properties of the Portuguese version has provided initial evidence for the validity of this measure, in which Cronbach’s alphas were as follows: overall well-being (0.92), negative emotion (0.71), and physical health (0.86) (Alves et al., 2016). In this sample, a Cronbach’s alpha of 0.94 was observed for the overall well-being subscale, 0.85 for perceived physical health, and 0.75 for negative emotion. Total scores of each subscale are calculated by summing the corresponding items, in which higher scores reflect greater levels of overall well-being, physical health and negative emotion.

#### Depression, Anxiety and Stress

The Depression, Anxiety and Stress Scale (DASS-21) was used in this study. This scale is a shorter version of 21 items of the original scale of 42 items scale developed by Lovibond and Lovibond (1995), which also showed acceptable internal consistency and concurrent validity (Antony et al., 1998). Antony et al. (1998) observed an alpha of 0.94 for Depression, 0.91 for Stress, and 0.87 for anxiety. This scale was translated and adapted to Portuguese by Pais-Ribeiro et al. (2004) and showed good psychometric properties, with Cronbach’s alphas of 0.85 for depression, 0.74 for anxiety, and 0.81 for stress. It intends to measure self-reported anxiety (e. g, “I was aware of dryness of my mouth.”) and depression (e.g., “I felt that I had nothing to look forward to”), as well as symptoms of stress, such as physical arousal, psychological tension and agitation (e.g., “I found it difficult to relax”). Each dimension is comprised of seven items answered on a 4-point scale ranging from 0 (*did not apply to me at all)* to 3 (*applied to me very much, or most of the time*). An alpha of 0.90 was obtained for stress; of 0.84 for anxiety; and 0.84 for depression. Scores were calculated by summing the items corresponding to each subscale and doubling the result up to allow the comparison with other international studies on COVID-19 (Rodríguez-Rey et al., 2020; Wang et al., 2020). Therefore, subscales scores can be interpreted according to different levels of severity (Lovibond and Lovibond, 1995; Wang et al., 2020). For anxiety, values were as follows: normal (0–6), mild (7–9), moderate (10–14), severe (15–19), and extremely severe (20–42). Stress could be interpreted according to the following scores: normal (0–10), mild (11–18), moderate (19–26), severe (27–34), and extremely severe (35–42). Lastly, depression severity levels were: normal (0–9), mild (10–12), moderate (13–20), severe (21–27), and extremely severe (28–42).

#### Coping

For this study, the Brief COPE (Carver, 1997) was used to assess coping strategies. This has already been translated and adapted to Portuguese (Pais-Ribeiro and Rodrigues, 2004; Dias et al., 2009). Previous Portuguese versions have found similar Cronbach’s alphas to those originally presented by Carver (1997) (0.54–0.90), ranging between 0.32–0.82 (Dias et al., 2009) and 0.55–0.84 (Pais-Ribeiro and Rodrigues, 2004). It includes 28 items (e.g., “I’ve been criticizing myself”) distributed by 14 subscales of two items each. Specifically, these subscales include: self-distraction, reflecting an attempt to do something not to think about the stressor; active coping, which refers to acting and making efforts to deal with the stressor; denial, reflecting attempts to avoid the reality of the stressful event; emotional support, describing actions of getting emotional support from others; instrumental support, including getting help, information, and advice about what to do from others; behavioral disengagement, which consists of giving up the attempt to attain the goals with which the stressor is interfering; venting, describing the tendency to focus on the stressor, expressing the negative feelings; positive reappraisal, consisting in reappraising the stressful transaction in positive terms; planning, such as plan efforts to cope with the situation; humor, reflecting the of use humor to deal with the stressor; religion, as in seeking spiritual and/or religious support; self-blame, consisting in criticizing oneself for the responsibility of the situation; substance abuse, reflecting the use of drugs or alcohol to deal with the stressor; and acceptance, consisting in accepting the situation. Participants were asked to answer on a Likert-type scale of 4 ranging from 1 (*I never do this*) to 4 (*I always do this*). Scores are calculated by summing the corresponding items of each subscale, in which higher scores reflect the tendency to use that coping strategy. In the current sample, alphas were as follows: Planning (0.48), Active coping (0.53), Denial (0.63), Substance abuse (0.74), Emotional support (0.70), Instrumental support (0.67), Behavioral disengagement (0.64), Venting (0.35), Positive reframing (0.77), Humor (0.76), Acceptance (0.72), Religion (0.82), Self-blame (0.43), and Distraction (0.49).

#### Experiential Avoidance

This construct was measured using the Acceptance and Action Questionnaire – II (AAQ-II), developed as a general measure of experiential avoidance and psychological inflexibility (Bond et al., 2011), which has showed good psychometric properties with alphas ranging from 0.78 to 0.88 across six samples. Overall, it comprises seven items (e.g., “I am afraid of my feelings”) reflecting an unwillingness to experience undesirable thoughts and emotions, as well as the inability to be in the present moment and facing negative psychological events by staying committed to flexible value-directed actions (Bond et al., 2011), answered on a 7-point scale ranging from 1 (*never true*) to 7 (*always true*). The Portuguese version has shown good psychometric properties and appropriate discriminant validity, with a Cronbach’s alpha of 0.92 (Costa et al., 2014). The AAQ-II also showed appropriate internal consistency (α = 0.93) in the current sample. The total score is obtained by summing all the items, in which higher levels reflect a greater tendency to avoid negative experiences.

#### Positivity

This study used the Positivity Scale, which was originally developed by Caprara et al. (2012) as a short measure of the tendency to “view life and experiences with a positive outlook” (p. 701). It includes a total of eight items (e.g., “I have great faith in the future”) rated on a 5-point Likert ranging from 1 (*strongly disagree*) to 5 (*strongly agree*). The original scale has showed appropriate validity with an alpha of 0.78 and of 0.79 in two different samples. Initial validation studies have found support for the Portuguese version (Cruz et al., 2016), which has revealed a Cronbach alpha of 0.75. In this sample, this scale showed good internal consistency (α = 0.86). Scores are calculated by summing all the items, in which greater values reflect higher levels of positivity.

### Procedures

Participants were invited to complete an online survey via social media and e-mail, in which they were informed of the goals of the study. Data were collected in the initial phase of the pandemic crisis in Portugal, more specifically during full lockdown months (April and May). All respondents were informed of the anonymous and voluntary nature of their participation, as well as the possibility of withdrawal at any moment. The full questionnaire opened with a cover letter, which included all these details, to which participants had to agree and consent before starting the completion. Participants did not receive any compensation for taking part in this study. Ethical approval was obtained from the Ethics and Deontology Commission for Scientific Research of Faculty of Psychology, Education and Sports, Lusófona University.

## Results

### Data Analytic Strategy

In order to identify the main predictors of psychological adjustment during the COVID-19 pandemic, descriptive statistics and Pearson correlation analyses were performed to explore the pattern of correlations. Subsequently, a hierarchical regression analysis was conducted to identify the main predictors of psychological adjustment during the COVID-19 pandemic (Tabachnick and Fidell, 2007). Separate hierarchical regressions were conducted for anxiety, depression, stress, well-being, physical health, loneliness and negative emotion, introducing sociodemographic variables at the first step (gender, age, and perceived social status), positivity and experiential avoidance at the second step and coping strategies at the third step.

As a measure of effect size, the *f* ^2^ of Cohen was computed, in which an effect of 0.02 is considered small, 0.15 medium, and 0.35 large (Cohen, 1988). After screening for multivariate outliers using the Mahalanobis distance, 18 outliers were identified and deleted from the regression analyses. Normality was assessed through skewness (Sk) and kurtosis (Ku), in which values bellow 3 and 10, respectively, were considered indicative of a normal distribution (Kline, 2009). The assumption of no multicollinearity was satisfied: correlations between the independent variables were not greater than 0.80, with values of variance inflation factor (VIF) below 10, and tolerance values above 0.2 (Hair et al., 2010). Values for the Durban–Watson statistic ranged between 1.92 and 2.08, thus were within the range of above 1 and below 3, which satisfies the assumption of independent errors. An α-level of 0.01 was used for considering the regression results statistically significant in order to control for α-inflation. All analyses were performed using the IBM SPSS version 26.

### Descriptive Statistics

Table 2 shows descriptive statistics of the variables in the study. Overall, the depression scale showed a mean score of 7.55 (*SD* = 7.54) for the total sample, in which 402 (68.6%) participants showed normal levels of depression, 70 (11.9%) mild depression, 68 (11.6%) moderate depression, 31 (5.3%) severe depression, and 15 (2.6%) extremely severe depression. When considering anxiety, a mean score of 5.37 was observed (*SD* = 7.21), distributed across the following levels of severity: normal (425, 72.5%); mild (38, 6.5%); moderate (55, 11.1%); severe (23, 3.9%); and extremely severe (35, 6%). Lastly, a mean score of 13.27 (*SD* = 9.86) was found for stress. A total of 265 participants (45.2%) were classified as showing normal levels of stress, 172 (29.4%), mild stress, 83 (14.2%) moderate stress, 45 (7.7%) severe stress, and 21 (3.6%) extremely severe stress.

**TABLE 2:** Descriptive statistics.

	*M*	*SD*	Min.	Max.
Anxiety	5.37	7.21	0.00	42.00
Depression	7.55	7.54	0.00	38.00
Stress	13.27	9.86	0.00	42.00
Well-being	4.38	0.93	0.63	5.94
Perceived physical health	4.16	1.09	0.00	6.00
Loneliness	1.99	1.82	0.00	6.00
Negative emotion	2.43	1.21	0.00	5.67
Positivity	3.76	.65	1.50	5.00
Experiential avoidance	19.05	9.11	7.00	49.00
Self-distraction	5.47	1.69	2.00	8.00
Active coping	6.18	1.40	2.00	8.00
Denial	2.49	1.06	2.00	8.00
Emotional support	4.27	1.73	2.00	8.00
Instrumental support	4.39	1.68	2.00	8.00
Behavioral disengagement	2.42	.98	2.00	8.00
Venting	4.66	1.56	2.00	8.00
Positive reframing	6.00	1.57	2.00	8.00
Planning	5.83	1.42	2.00	8.00
Humor	4.92	1.77	2.00	8.00
Religion	3.88	1.90	2.00	8.00
Self-blame	3.11	1.27	2.00	8.00
Substance abuse	2.21	.71	2.00	8.00
Acceptance	6.66	1.35	2.00	8.00

*Anxiety, depression, and stress scores were calculated by summing the items corresponding to each subscale and doubling the result up to allow the comparison with other international studies on COVID-19.*

An overall inspection of the mean scores for coping strategies revealed that acceptance (*M* = 6.66, *SD* = 1.35), active coping (*M* = 6.18, *SD* = 1.40), and positive reframing (*M* = 6.00, *SD* = 1.55) showed the highest mean scores whereas substance abuse (*M* = 2.21, *SD* = 0.71), behavioral disengagement (*M* = 2.42, *SD* = 0.98), and denial (*M* = 2.49, *SD* = 1.06) showed the lowest scores.

### Pearson Correlations

The patterns of associations between the variables in the study can be found in Table 3. Generally, positivity seems to be associated with more positive outcomes, such as well-being and physical health, and less negative outcomes, including anxiety, depression, stress, negative emotion, and loneliness. Consistently, experiential avoidance was negatively associated with measures of psychological adjustment (well-being and perceived physical health), and positively with psychological distress (anxiety, stress, depression), negative emotion, and loneliness.

**TABLE 3:** Pearson correlations.

	1	2	3	4	5	6	7	8	9	10	11	12
(1) Anxiety	1.00	0.65^∗∗∗^	0.73^∗∗∗^	−0.34^∗∗∗^	−0.40^∗∗∗^	0.22^∗∗∗^	0.49^∗∗∗^	−0.35^∗∗∗^	0.50^∗∗∗^	0.20^∗∗∗^	−0.03	0.31^∗∗∗^
(2) Depression		1.00	0.71^∗∗∗^	−0.54^∗∗∗^	−0.41^∗∗∗^	0.33^∗∗∗^	0.57^∗∗∗^	−0.49^∗∗∗^	0.57^∗∗∗^	0.19^∗∗∗^	−0.18^∗∗∗^	0.23^∗∗∗^
(3) Stress			1.00	−0.32^∗∗∗^	−0.35^∗∗∗^	0.27^∗∗∗^	0.65^∗∗∗^	−0.36^∗∗∗^	0.54^∗∗∗^	0.24^∗∗∗^	0.00	0.19^∗∗∗^
(4) Well-being				1.00	0.54^∗∗∗^	−0.34^∗∗∗^	−0.32^∗∗∗^	0.79^∗∗∗^	−0.49^∗∗∗^	0.01	0.36^∗∗∗^	−0.12^∗∗^
(5) Perceived physical health					1.00	−0.25^∗∗∗^	−0.30^∗∗∗^	0.50^∗∗∗^	−0.34^∗∗∗^	−0.05	0.17^∗∗∗^	−0.11^∗∗^
(6) Loneliness						1.00	0.38^∗∗∗^	−0.42^∗∗∗^	0.45^∗∗∗^	0.17^∗∗∗^	−0.05	0.11^∗∗^
(7) Negative emotion							1.00	−0.42^∗∗∗^	0.61^∗∗∗^	0.22^∗∗∗^	−0.06	0.24^∗∗∗^
(8) Positivity								1.00	−0.60^∗∗∗^	−0.10^∗^	0.26^∗∗∗^	−0.13^∗∗^
(9) Experiential avoidance									1.00	0.21^∗∗∗^	−0.09^∗^	0.30^∗∗∗^
(10) Self-distraction										1.00	0.19^∗∗∗^	0.19^∗∗∗^
(11) Active coping											1.00	0.07
(12) Denial												1.00

		13	14	15	16	17	18	19	20	21	22	23

(1) Anxiety		0.13^∗∗∗^	0.18^∗∗∗^	0.32^∗∗∗^	0.21^∗∗∗^	−0.11^∗∗^	0.00	−0.10^∗^	0.08^∗^	0.33^∗∗∗^	0.09^∗^	−0.21^∗∗∗^	
(2) Depression		0.11^∗∗^	0.09^∗^	0.46^∗∗∗^	0.11^∗∗^	−0.28^∗∗∗^	−0.08^∗^	−0.11^∗^	0.00	0.42^∗∗∗^	0.27^∗∗∗^	−0.27^∗∗∗^	
(3) Stress		0.24^∗∗∗^	0.24^∗∗∗^	0.29^∗∗∗^	0.25^∗∗∗^	−0.16^∗∗∗^	0.04	−0.04	0.06	0.37^∗∗∗^	0.17^∗∗∗^	−0.21^∗∗∗^	
(4) Well-being		0.16^∗∗∗^	0.15^∗∗∗^	−0.36^∗∗∗^	0.13^∗∗^	0.45^∗∗∗^	0.35^∗∗∗^	0.22^∗∗∗^	0.15^∗∗∗^	−0.26^∗∗∗^	−0.21^∗∗∗^	0.40^∗∗∗^	
(5) Perceived physical health		0.01	0.02	−0.24^∗∗∗^	0.01	0.25^∗∗∗^	0.18^∗∗∗^	0.11^∗∗^	0.07	−0.25^∗∗∗^	−0.13^∗∗^	0.32^∗∗∗^	
(6) Loneliness		0.03	0.02	0.23^∗∗∗^	0.06	−0.13^∗∗^	−0.02	−0.01	0.00	0.27^∗∗∗^	0.10^∗^	−0.11^∗∗^	
(7) Negative emotion		0.15^∗∗∗^	0.16^∗∗∗^	0.29^∗∗∗^	0.14^∗∗^	−0.22^∗∗∗^	0.00	−0.13^∗∗^	0.06	0.37^∗∗∗^	0.15^∗∗∗^	−0.22^∗∗∗^	
(8) Positivity		0.08	0.06	−0.36^∗∗∗^	0.06	0.38^∗∗∗^	0.24^∗∗∗^	0.18^∗∗∗^	0.17^∗∗∗^	−0.28^∗∗∗^	−0.19^∗∗∗^	0.34^∗∗∗^	
(9) Experiential avoidance		0.11^∗∗^	0.15^∗∗∗^	0.35^∗∗∗^	0.15^∗∗∗^	−0.20^∗∗∗^	−0.07	−0.12^∗∗^	0.04	0.41^∗∗∗^	0.20^∗∗∗^	−0.28^∗∗∗^	
(10) Self-distraction		0.19^∗∗∗^	0.20^∗∗∗^	0.12^∗∗^	0.21^∗∗∗^	0.04	0.17^∗∗∗^	0.13^∗∗^	0.11^∗∗^	0.10^∗^	0.03	0.06	
(11) Active coping		0.20^∗∗∗^	0.24^∗∗∗^	−0.16^∗∗∗^	0.31^∗∗∗^	0.43^∗∗∗^	0.53^∗∗∗^	0.23^∗∗∗^	0.18^∗∗∗^	0.04	−0.03	0.34^∗∗∗^	
(12) Denial		0.01	0.11^∗∗^	0.27^∗∗∗^	0.12^∗∗^	0.00	−0.03	−0.06	0.11^∗∗^	0.14^∗∗^	0.03	−0.22^∗∗∗^	
(13) Emotional Support		1.00	0.75^∗∗∗^	0.04	0.39^∗∗∗^	0.20^∗∗∗^	0.28^∗∗∗^	0.22^∗∗∗^	0.18^∗∗∗^	0.18^∗∗∗^	0.10^∗^	0.11^∗∗^	
(14) Instrumental Support			1.00	0.07	0.45^∗∗∗^	0.22^∗∗∗^	0.31^∗∗∗^	0.21^∗∗∗^	0.23^∗∗∗^	0.17^∗∗∗^	0.09^∗^	0.11^∗∗^	
(15) Behavioral disengagement				1.00	0.03	−0.16^∗∗∗^	−0.12^∗∗^	−0.05	0.01	0.28^∗∗∗^	0.23^∗∗∗^	−0.23^∗∗∗^	
(16) Venting					1.00	0.35^∗∗∗^	0.38^∗∗∗^	0.23^∗∗∗^	0.28^∗∗∗^	0.17^∗∗∗^	0.08^∗^	0.23^∗∗∗^	
(17) Positive reframing						1.00	0.46^∗∗∗^	0.40^∗∗∗^	0.23^∗∗∗^	−0.01	−0.10^∗^	0.49^∗∗∗^	
(18) Planning							1.00	0.20^∗∗∗^	0.24^∗∗∗^	0.09^∗^	0.01	0.42^∗∗∗^	
(19) Humor								1.00	−0.01	0.07	−0.02	0.31^∗∗∗^	
(20) Religion									1.00	0.07	−0.04	0.10^∗^	
(21) Self-blame										1.00	0.20^∗∗∗^	−0.09^∗^	
(22) Substance abuse											1.00	−0.05	
(23) Acceptance												1.00	

*p < 0.05. **p < 0.01. ***p < 0.001.

Analyses of coping strategies suggest that behavioral disengagement and self-blame were consistently associated with poorer psychological outcomes. Conversely, positive reframing and acceptance were associated with more positive outcomes in terms of psychological adjustment.

### Predictors of Psychological Distress and Well-Being

With regards to anxiety, gender contributed significantly to the regression model, *F*(4,563) = 8.12, *p* < 0.001, and accounted for 6% of the variation in anxiety. At the second step, experiential avoidance contributed to the model, *F*(6,561) = 34.53, *p* < 0.001, explaining 22% of the variance, with a significant change, *F*(2,561) = 82.66, *p* < 0.001(Cohen’s *f*^2^ = 0.28). The third step showed that denial, behavioral disengagement and venting were predictors of anxiety, *F*(20,547) = 15.17, *p* < 0.001, and accounted for 9% of the variance, also with a significant change, *F*(14,547) = 5.29, *p* < 0.001 (Cohen’s *f*^2^ = 0.14) (see Table 4).

**TABLE 4:** Hierarchical regression analyses for predicting psychological distress.

	Anxiety				Depression					Stress		
						
	*B*	*SE*	β		*B*	*SE*	β			*B*	*SE*	β
**Step 1**	(*R^2^* = 0.06; Δ*R*^2^ = 0.05)				(*R^2^* = 0.06; Δ*R*^2^ = 0.06)					(*R^2^* = 0.11; Δ*R*^2^ = 0.11)		
Gender	−2.82	0.67	−0.18^∗∗∗^		−2.47	0.67	−0.15^∗∗∗^			−5.41	0.88	−0.25^∗∗∗^
Age	−0.04	0.02	−0.07		−0.07	0.02	−0.12^∗∗^			−0.13	0.03	−0.17^∗∗∗^
High social status	−1.16	0.97	−0.05		−1.29	0.97	−0.06			−1.05	1.28	−0.03
Low social status	1.99	1.11	0.07		1.74	1.11	0.07			0.22	1.46	0.01
**Step 2**	(*R^2^* = 0.27; Δ*R*^2^ = 0.22)				(*R^2^* = 0.35; Δ*R*^2^ = 0.29)					(*R^2^* = 0.35; Δ *R*^2^ = 0.24)		
Positivity	−0.85	0.52	−0.08		−2.8	0.49	−0.25^∗∗∗^			−1.23	0.66	−0.08
Experiential avoidance	0.35	0.04	0.43^∗∗∗^		0.29	0.04	0.36^∗∗∗^			0.50	0.05	0.45^∗∗∗^
**Step 3**	(*R^2^* = 0.36; Δ*R*^2^ = 0.09)				(*R^2^* = 0.46; Δ*R*^2^ = 0.11)					(*R^2^* = 0.42; Δ*R*^2^ = 0.07)		
Self-distraction	0.25	0.16	0.06		0.22	0.15	0.05			0.35	0.21	0.06
Active coping	−0.09	0.22	−0.02		−0.47	0.20	−0.09			0.15	0.28	0.02
Denial	1.08	0.28	0.15^∗∗∗^		0.31	0.26	0.04			0.21	0.36	0.02
Emotional support	−0.11	0.22	−0.03		0.21	0.20	0.05			0.34	0.28	0.06
Instrumental support	0.27	0.23	0.06		−0.28	0.21	−0.07			0.15	0.30	0.03
Behavioral disengagement	1.05	0.33	0.12^∗∗∗^		1.64	0.30	0.19^∗∗∗^			0.97	0.42	0.09
Venting	0.61	0.20	0.13^∗∗∗^		0.38	0.18	0.08			0.81	0.26	0.13^∗∗^
Positive reframing	−0.11	0.22	−0.03		−0.65	0.20	−0.14^∗∗^			−0.85	0.28	−0.14^∗∗^
Planning	−0.08	0.23	−0.02		0.17	0.21	0.03			0.21	0.30	0.03
Humor	−0.14	0.16	−0.03		0.01	0.15	0.00			0.20	0.21	0.04
Religion	0.04	0.14	0.01		0.04	0.13	0.01			−0.05	0.19	−0.01
Self-blame	0.51	0.23	0.09		0.95	0.21	0.16^∗∗∗^			0.78	0.30	0.10^∗∗^
Substance abuse	−0.17	0.49	−0.01		1.07	0.45	0.08			0.41	0.62	0.02
Acceptance	−0.28	0.24	−0.05		−0.15	0.22	−0.03			−0.57	0.30	−0.08

	**Well being**			**Well being**			**Loneliness**			**Negative emotion**		
				
	***B***	***SE***	**β**	***B***	***SE***	**β**	***B***	***SE***	**β**	***B***	***SE***	**β**

**Step 1**	(*R^2^* = 0.03; Δ*R*^2^ = 0.03)			(*R^2^* = 0.02; Δ*R*^2^ = 0.02)			(*R^2^* = 0.01; Δ*R*^2^ = 0.01)			(*R^2^* = 0.06; Δ*R*^2^ = 0.06)		
Gender	0.02	0.09	0.01	0.25	0.10	0.10	−0.15	0.17	−0.04	−0.42	0.11	−0.16^∗∗∗^
Age	0.00	0.00	0.02	−0.00	0.00	−0.03	0.00	0.01	0.03	−0.01	0.00	−0.12^∗∗^
High social status	0.34	0.13	0.11^∗∗^	0.24	0.15	0.07	−0.24	0.25	−0.04	−0.17	0.16	−0.04
Low social status	−0.34	0.14	−0.10^∗∗^	−0.17	0.17	−0.04	0.45	0.29	0.07	0.24	0.18	0.05
**Step 2**	(*R^2^* = 0.61; Δ*R*^2^ = 0.58)			(*R^2^* = 0.24; Δ*R*^2^ = 0.22)			(*R^2^* = 0.22; Δ*R*^2^ = 0.22)			(*R^2^* = 0.39; Δ*R*^2^ = 0.33)		
Positivity	1.11	0.05	0.77^∗∗∗^	0.76	0.08	0.44^∗∗∗^	−0.68	0.13	−0.24^∗∗∗^	−0.23	0.08	−0.12^∗∗^
Experiential avoidance	−0.00	0.00	−0.01	−0.01	0.01	−0.06	0.06	0.01	0.29^∗∗∗^	0.07	0.01	0.51^∗∗∗^
**Step 3**	(*R^2^* = 0.67; Δ*R*^2^ = 0.06)			(*R^2^* = 0.28; Δ*R*^2^ = 0.05)			(*R^2^* = 0.25; Δ*R*^2^ = 0.02)			(*R^2^* = 0.43; Δ*R*^2^ = 0.04)		
Self-distraction	0.04	0.02	0.07	−0.01	0.03	−0.01	0.07	0.04	0.07	0.05	0.03	0.07
Active coping	0.05	0.02	0.07	0.01	0.04	0.01	0.00	0.06	0.00	−0.02	0.03	−0.03
Denial	−0.01	0.03	−0.01	−0.04	0.05	−0.03	−0.05	0.08	−0.03	0.04	0.04	0.03
Emotional support	0.04	0.02	0.07	−0.04	0.04	−0.02	0.07	0.06	0.06	0.02	0.03	0.03
Instrumental support	0.01	0.02	0.05	0.01	0.04	0.02	−0.09	0.06	−0.08	0.01	0.04	0.01
Behavioral disengagement	−0.07	0.03	−0.06	0.00	0.05	0.00	0.06	0.09	0.03	0.07	0.05	0.05
Venting	−0.00	0.02	−0.01	−0.03	0.03	−0.04	0.01	0.05	0.01	0.02	0.03	0.03
Positive reframing	0.04	0.02	0.07	0.02	0.04	0.03	−0.03	0.06	−0.03	−0.10	0.03	−0.13^∗∗^
Planning	0.06	0.02	0.09^∗∗^	0.03	0.04	0.04	0.04	0.06	0.03	0.05	0.04	0.06
Humor	−0.01	0.02	−0.01	−0.02	0.03	−0.03	0.06	0.04	0.06	−0.04	0.03	−0.06
Religion	−0.01	0.01	−0.02	0.01	0.02	0.02	0.01	0.04	0.02	0.02	0.02	0.03
Self-blame	−0.03	0.02	−0.04	−0.10	0.04	−0.11^∗∗^	0.07	0.06	0.04	0.11	0.04	0.11^∗∗^
Substance abuse	−0.06	0.04	−0.04	0.04	0.08	0.02	−0.13	0.13	−0.04	0.04	0.08	0.02
Acceptance	0.04	0.02	0.06	0.14	0.04	0.18^∗∗∗^	0.06	0.06	0.04	0.01	0.04	0.01

Categorical variables were coded as follows: 0 = females and 1 = males; 1 = High social status, 0 = Low and medium social status; 1 = Low social status, 0 = High and medium social status. **p < 0.01. ***p < 0.001.

In analysis of the predictors of depression, gender and age were significant predictors in the first step, *F*(4,563) = 9.04, *p* < 0.001, explaining 6% of the variance. Subsequently, experiential avoidance and positivity explained 29% of the variance, *F*(6,561) = 49.30, *p* < 0.001, with a significant change in the model, *F*(2,561) = 122.05, *p* < 0.001 (Cohen’s *f*^2^ = 0.45). In the third step, behavioral disengagement, self-blame, and positive reframing were significant with a variance explained of 11%, *F*(20,547) = 23.02, *p* < 0.001, significantly contributing to the change in the model, *F*(14,547) = 8.05, *p* < 0.001 (Cohen’s *f*^2^ = 0.20).

When using this model to predict stress, gender and age were significant predictors in the first step, *F*(4,563) = 17.02, *p* < 0.001, and explained 11% of the variance. The second step explained 24%, *F*(6,561) = 50.26, *p* < 0.001, with experiential avoidance significantly contributing to the model, *F*(2,561) = 104.23, *p* < 0.001 (Cohen’s *f*^2^ = 0.37). The introduction of coping strategies at the third step demonstrated that positive reframing, venting, and self-blame were significant predictor of stress, *F*(20,547) = 19.86, *p* < 0.001 (7% of the variance explained, *F*(14,547) = 4.79, *p* < 0.001, Cohen’s *f*^2^ = 0.12).

In the analysis of well-being, high socioeconomic status was a significant predictor in the first step, *F*(4,563) = 3.84, *p* = 0.004 (3% variance explained). The inclusion of the variables in the second step suggested that positivity was a significant predictor, *F*(6,561) = 146.05, *p* < 0.001 (58% variance explained), with a significant change in the model, *F*(2,561) = 419.09, *p* < 0.001 (Cohen’s *f*^2^ = 1.49). The final step revealed that planning significantly contributed to the model, *F*(20,547) = 56.54, *p* < 0.001, and increased the variance explained with an additional 6%, *F*(14,547) = 7.70, *p* < 0.001 (Cohen’s *f*^2^ = 0.18).

Perceived physical health was not predicted by sociodemographic variables, *F*(4,563) = 2.68, *p* = 0.031. In the second step, positivity was a significant predictor, *F*(6,561) = 29.12, *p* < 0.001, explaining 22% of variance explained, *F*(2,561) = 80.48, *p* < 0.001 (Cohen’s *f*^2^ = 0.29). When adding coping strategies, acceptance and self-blame were significant predictors, *F*(20,547) = 10.76, *p* < 0.001, accounting for 5% of the variance, *F*(14,547) = 2.44, *p* < 0.001 (Cohen’s *f*^2^ = 0.06).

The introduction of the sociodemographic variables in the first suggested that none of the variables were significant predictors of loneliness, *F*(4,563) = 1.19, *p* = 0.315. However, experiential avoidance and positivity were significant predictors, *F*(6,561) = 26.87, *p* < 0.001, explaining 22% of the variance in loneliness, *F*(2,561) = 77.59, *p* < 0.001 (Cohen’s *f*^2^ = 0.24). The third step did not produce a significant change, *F*(14,547) = 1.14, *p* = 0.323, in which none of the coping strategies were significant predictors of loneliness.

Finally, gender and age were predictors of negative emotion in the first step, *F*(4,563) = 8.87, *p* < 0.001 (6% of explained variance). Experiential avoidance and positivity were significant predictors in the second step, *F*(6,561) = 58.81, *p* < 0.001, and explained 33% of the variance, *F*(2,561) = 150.77, *p* < 0.001 (Cohen’s *f*^2^ = 0.54). The introduction of coping strategies revealed that positive reframing and self-blame were significant predictors, *F*(20,547) = 20.45, *p* < 0.001, accounting for 4% of the variance, *F*(14,547) = 2.85, *p* < 0.001 (Cohen’s *f*^2^ = 0.07).

## Discussion

This study, framed within the scope of Existential Positive Psychology (PP2.0), aimed at assessing the levels of psychological distress of adults living in Portugal during the first national lockdown associated with the COVID-19 outbreak and how they are coping with the stress associated with the pandemic crisis. It also intended to examine the association between positivity, experiential avoidance, and coping responses used during the national lockdown with self-reported well-being and psychological distress, namely, depression, anxiety, and stress.

Concerning psychological distress, participants in the current study reported 19.5, 21, and 25.5% of moderate to extremely severe symptoms of depression, anxiety, and stress, respectively. Overall, these results are higher than those reported in previous COVID-19 Portuguese studies (Paulino et al., 2020; Moreira et al., 2021), especially for stress and anxiety. This difference may be related to data collection timing. While the current study collected the data during April and May, the two previous studies collected their data only 4 or 5 days after the beginning of the first national lockdown. In addition to various other dispositional and situational aspects, the psychological response to a stressor closely depends on the duration over which the individual is exposed to it (Lazarus, 1998). Although it took place during the confinement period, the data collection for this study started only 1 month after it began, allowing us to analyze the participants’ responses after some length of exposure to the stressor.

Findings from the current study, however, appear relatively lower than those observed in the Spanish population for anxiety (24%) and depression (30%) but higher for stress (22%) (Rodríguez-Rey et al., 2020). Several other studies have examined mental health and psychological distress indicators in the population affected by the pandemic crisis and the national authorities’ lockdowns. Some of these studies have concluded that this global COVID-19 pandemic crisis is causing unprecedented negative psychological consequences (Brooks et al., 2020; Rodríguez-Rey et al., 2020; Wang et al., 2020), while others have not suggested such impact (Jarego et al., 2021). However, comparisons with other studies, even when data collections are conducted relatively within the same temporal window, have to be made carefully. It is essential to keep in mind that pandemic evolution is very diverse from country to country and often even from region to region within the same country, as are the pandemic mitigation responses that affect individuals’ lives. For example, Portugal was more severely affected by the COVID-19 pandemic later comparing with other European countries. Besides, containing measures were taken relatively sooner, as other countries’ experience guided some Portuguese governmental decisions. These circumstances and the perceived relative success of containment measures in the pandemic’s progression make these comparisons very difficult and may help understand the variability of results found in the literature. Nevertheless, the findings seem consistent concerning the pandemic crisis’s distinct impact on different population groups, specifically regarding gender and age. In line with previous studies (González-Sanguino et al., 2020; Paulino et al., 2020; Pieh et al., 2020; Rodríguez-Rey et al., 2020; Wang et al., 2020; Wanberg et al., 2020), the current study also observed higher levels of psychological distress in females and younger adults. Indeed, the pandemic may primarily affect women by potentially increasing their burden at home, as they are usually informal family caregivers (Mantovani et al., 2020; Rodríguez-Rey et al., 2020). Conversely, older adults seem to have a more optimistic outlook during the pandemic’s initial stages (e.g., Bruine de Bruin, 2020), which appears to explain why the younger population is particularly affected. It is also possible that more younger people found themselves more limited in the activities they usually did than older people. Besides, the literature has indicated that younger people tend to have more difficulty dealing with solitude than older people (Larson et al., 1985; Lay et al., 2018).

One of the main aims of this study was to examine the associations between the dispositional variables of experiential avoidance and positivity with the participants’ levels of psychological distress and well-being. Results showed that higher levels of experiential avoidance predict adverse effects of COVID-19 pandemic crises in terms of psychological distress and negative emotions, consistently with findings from prior research, suggesting that experiential avoidance is related to depression and anxiety (Zvolensky et al., 2016; Moroz and Dunkley, 2019). Recent COVID-19 specific research has also demonstrated that psychological inflexibility exacerbates the detrimental impacts of COVID-19 on mental health (Dawson and Golijani-Moghaddam, 2020; Kroska et al., 2020; Pakenham et al., 2020) and psychological adjustment (Seçer et al., 2020).

Conversely, a more positive response, as measured by physical, emotional, and psychological well-being, seems to be better predicted by the dispositional tendency to have a positive outlook on life experiences (positive life orientation or positivity). Positivity is the strongest predictor of well-being, as well as an essential protective factor for depression. This finding was consistent with previous studies that found positivity as a protective factor against mental illness, in general (Alessandri et al., 2012b; Caprara et al., 2017, 2018) and as a critical dimension for positive adaptation in the current pandemic situation (Trzebiński et al., 2020; Yıldırım and Güler, 2021).

Regarding coping strategies, in a first analysis of the descriptive results, it was observed that the coping strategies most used among the participants of the current study to deal with the ongoing pandemic crisis were acceptance, active coping, and positive reframing. In line with Existential Positive Psychology, these strategies reflect a dialectical way of coping with life demands, accepting the dark aspects of one’s life, embracing them in a positive direction, and responding proactively to a specific problematic situation (see Wong et al., 2006). Alternatively, substance abuse, behavioral disengagement, and denial were the least reported. Similar findings were also reported in Jarego et al. (2021) study, conducted in Portugal during the same period, in the first overall lockdown, who suggested that social desirability might explain the low levels of substance abuse. Additionally, denial was also reported in their study as a less commonly used strategy to deal with the current crisis. Consistently with their sample, participants in the present study were also highly educated, which may lead to more access to information and thus less use of this strategy (Jarego et al., 2021). Nonetheless, substance abuse and behavioral disengagement were also the least commonly used strategies to deal with the stress associated with the pandemic in an American sample (Park et al., 2020).

Furthermore, findings from the current study provide important evidence for coping strategies associated with better or poor adjustment to the current pandemic crisis. Overall, findings highlighted three coping strategies that appear to be significant risk factors: self-blame, behavioral disengagement, and emotional venting. More specifically, behavioral disengagement was a predictor of anxiety and depression; venting was a significant predictor of anxiety and stress; and self-blame was a predictor of depression, stress, and negative emotions. It was also observed that denial constituted a risk factor since it predicted anxiety. Indeed, previous research consistently shows that avoidance or disengagement coping is associated with poor outcomes (Babore et al., 2020; Mariani et al., 2020; Rettie and Daniels, 2020).

Nevertheless, whereas behavioral disengagement, denial, and substance abuse were the least used by the participants, self-blame and venting were more widely used to deal with the de pandemic crisis, often related to a more detrimental psychological impact (Riolli and Savicki, 2010). Self-blame, specifically, appears to be particularly maladaptive in this pandemic crisis context (Shamblaw et al., 2021), which is characterized by uncertainty, unpredictability, and a decreased ability of individuals to act on the situation actively. As opposed to the catharsis theory, which suggests that emotional expression is psychologically beneficial, venting as a coping strategy seems to have amplified the job demands’ effects on psychological distress during the COVID-19 pandemic (Ben-Ezra and Hamama-Raz, 2020). Arguably, emotional ventilation by itself does not change the experience or its meaning. On the contrary, persistent expression of negative emotions can intensify damaging interpretations of the situation and keep the individual trapped in this dysfunctional process. Another potential reason behind the positive association between venting and distress is that higher distress levels can lead people to express their emotions to others as a regulation mechanism. Future experimental studies should test the causal relationship among these variables.

On the other hand, the positive reframing strategy was a strong predictor of low negative emotions while simultaneously appearing to be a protective factor for depression and stress. Planning has also been found to significantly predict well-being, while the coping strategy of acceptance was the strongest predictor of physical well-being (e.g., Polizzi et al., 2020; Zacher and Rudolph, 2020). Positive reframing, along with acceptance and humor, is part of a set of coping strategies designated by some authors as accommodative coping (e.g., Carver and Connor-Smith, 2010). In Jarego et al. (2021) study, only positive reframing and humor predicted better mental health. In the current study, humor did not predict well-being or psychological distress. Indeed, the empirical literature on the effects of humor is surprisingly inconclusive (see Samson and Gross, 2012), with a different set of consequences depending on the type of humor individuals use. Because the coping measure used to assess humor did not include this distinction, it was not possible to further explore these findings. As previously described, each coping strategy’s success essentially depends on the characteristics of the context and the control that the individual believes to have toward the stressor and the situation (Lazarus and Folkman, 1984). In the present crisis scenario, most circumstances are outside the individual’s control, decreasing the potential effectiveness of those strategies more oriented to deal directly with the stressor. This may help explain why active coping, although one of the most commonly used strategies by participants, has not been shown to be a significant predictor of well-being. Under these conditions, accommodative coping strategies, such as positive reframing and acceptance, are more successful. Interestingly, these were among the three strategies most frequently reported by the participants of this study to deal with the ongoing pandemic situation.

Contrary to other studies (e.g., Zacher and Rudolph, 2020), emotional and instrumental support and religion were not significantly associated with the well-being or psychological distress. This finding may be related to how some of the outcome measures were assessed. In the present study, overall well-being was assessed through positive emotions, involvement, relationships, meaning, and accomplishment, while in other studies, it was assessed, for example, only through life satisfaction. The awareness that everyone is “in the same boat” may also help explain why emotional and instrumental support does not predict well-being. With everyone potentially dealing with similar difficulties and challenges, it may have diminished individuals’ confidence in others’ ability to be an effective source of help and support. Lazarus (1993), while summarizing some of the essential conclusions about coping studies, suggested that despite the stability of the use of some strategies, others are highly context-dependent. Positive reappraisal, for example, has proven to be stable and more associated with dispositional characteristics, while seeking social support is unstable and considerably situational.

Regarding loneliness, no coping strategy was significantly associated with this negative subjective experience. The two great predictors were positivity as a protective factor, and experiential avoidance as a risk factor. Loneliness is considered a severe public health issue associated with an increased risk of morbidity and mortality even before the coronavirus crisis (Cacioppo and Cacioppo, 2018). In the present situation, where measures to reduce interpersonal contacts and social distance prevail, there is a growing concern that these measures may increase feelings of loneliness, especially among vulnerable groups (American Psychological Association, 2020). Previous research findings proposed that experiential avoidance or inflexibility can be seen as an essential underlying mechanism with significant associations with loneliness (Frinking et al., 2020). This study also highlights the positive and helpful contribution that a positive life orientation can play.

As was described earlier, positivity also plays a crucial role in predicting well-being and physical health. In contrast, experiential avoidance plays a more prominent part in predicting negative emotionality, stress, anxiety, and depression.

Concerning the contribution of coping strategies to the explained variance of the prediction models, it was found that coping strategies, in general, seem to contribute significantly more to the prediction of psychological distress than to well-being. This is consistent with the idea that coping strategies refer to individuals’ behavioral and cognitive efforts to reduce the pressure of a stressful situation when its demands exceed personal resources (Lazarus and Folkman, 1984). However, results also show that the use of strategies usually considered less adaptive (e.g., self-blaming, behavioral disengagement, ventilation, and denial) seem to be more significantly associated with negative functioning whereas strategies usually considered more positive seem to be associated with positive functioning. This finding raises the need for future studies to address the long-term impact of using these distinct strategies on psychological functioning, as well as focus on their effectiveness.

These results highlight the importance of studying several psychological variables in an integrated way and analyzing their specific contribution to well-being and psychological distress. The analysis of these two outcome measures allowed us to understand the variables that best predict a better/poorer psychological functioning.

Moreover, these findings may help health professionals design their psychological interventions in a more structured and targeted way. They also highlighted the need to develop and implement distinct interventions to promote well-being from those aimed at reducing individuals’ levels of psychological distress. The development of programs to improve mental health should be a priority in the government’s response to this pandemic crisis. Nevertheless, it is also essential that these programs are driven by the extensive scientific evidence that studies in this field have recently brought to light.

Notwithstanding these important contributions, the present study has some limitations that could be addressed in future research and considered when interpreting these findings. One first limitation concerns convenience sampling and data collection procedures. All data were collected via an online survey with self-report measures, and even though there is variety in the distributions of age, gender, and perceived socioeconomic status, the sample is not representative of the general Portuguese population. Findings may not be generalizable, and it is crucial to keep in mind that the data collection strategy may have excluded participants from socially and economically vulnerable groups, as well as older participants. This convenient sample is also composed mainly of highly educated participants. Another possible limitation concerns the low alpha values of some Brief COPE sub-scales, specifically venting, self-blame and planning. It is, therefore, necessary to read the results concerning these strategies more carefully. However, it was decided to keep all subscale in data analyses and discuss their results considering that the alpha value is affected by the number of items that compose each subscale. It is not expected that sub-scales with only two items have the same internal consistency values as longer or non-abbreviated scales (Streiner, 2003; Ziegler et al., 2014; Kato, 2015). Another limitation refers to the study design, in which the current cross-sectional design does not allow the examination of causal links among the variables over time. Finally, it was not possible to include all potentially relevant control variables, for instance, income loss, work, family demands, living conditions, or degree of relational satisfaction with co-habitants.

Therefore, it seems essential that future studies adopt a longitudinal design that allows us to go beyond the associations between the variables studied and understand some of the effects of the pandemic crisis on individuals’ psychological distress and well-being. Besides, it seems essential to investigate the impact of longer and shorter exposure to confinement, analyzing the moderating effect of the contextual conditions in which this confinement takes place. Studies using real-time data collection methods, within a more ecological perspective, could be useful to examine and understand the influence of the complex and dynamic interplay between individuals, coping strategies, and the contexts in which they are embedded (places, activities, and companies). Additionally, future studies should adopt data collection methods that allow access to the most disadvantaged groups in the population, as well as older people, who do not usually participate in online data collection. Future investigations should also attempt to explore the potential interaction between experiential avoidance and positivity on psychological adjustment. This would allow us to further uncover how the interplay between these dispositional traits affect well-being and psychological distress when facing stressful encounters.

## Conclusion

In line with Existential Positive Psychology, these findings appear to point out several psychological markers that help predict a better psychological adjustment among individuals’ responses toward this global threat. Results highlight the positive role of accepting painful experiences and emotions on individuals’ psychological distress and well-being, instead of the negative function of avoiding confrontation with suffering. When people are inquired about their lives’ great goal, a substantial part responds: being happy. This search for happiness can frequently turn out to be risky, especially when attempting to avoid any negative experience and emotion. Gruber et al. (2011) published a review suggesting that happiness pursuit and experience might sometimes lead to adverse outcomes: the dark side of happiness. They argue that an excessive degree of happiness manifested as an intensified level of the positive and relative absence of negative emotions can lead to undesirable outcomes. When people have to face unwanted situations, negative emotions, such as anger, fear, and sadness, may provide essential benefits that positive emotions do not, increasing their readiness to deal with the situation. On the contrary, engaging in the psychological flexibility processes increases resilience when facing adversity (Pakenham et al., 2020).

Experiential avoidance and positivity, along with specific coping strategies, represent promising candidates for understanding and predicting how individuals may be affected by multiple challenges of this pandemic and possibly other serious threats. These results indicate that psychological interventions targeting these malleable and responsive processes are likely to mitigate some adverse effects of the COVID-19 pandemic crisis in individual’s functioning. Additionally, findings also suggest the critical role of positive life orientations in promoting several components of well-being. This study provides essential and valuable cues for psychological interventions to promote a more positive and adaptive human functioning by better understanding the complex nature of the interaction between positive and negative life features.

## Data Availability Statement

The raw data supporting the conclusions of this article will be made available by the authors, without undue reservation.

## Ethics Statement

The studies involving human participants were reviewed and approved by Ethics Committee for the Scientific Research of Faculty of Psychology, Education and Sports, Lusófona University. The patients/participants provided their written informed consent to participate in this study.

## Author Contributions

MF, RS, JC, DC, and NE conceptualized and designed the study. MF, RS, and JC were involved in data collection. MF and RS prepared the first draft of the manuscript. JC, DC, NE, and IJ critically revised the draft and made important contributions, which improved the overall quality of the manuscript. All the authors have read and approved the final manuscript.

## Conflict of Interest

The authors declare that the research was conducted in the absence of any commercial or financial relationships that could be construed as a potential conflict of interest.
